# A novel microRNA promotes coxsackievirus B4 infection of pancreatic β cells

**DOI:** 10.3389/fimmu.2024.1414894

**Published:** 2024-12-04

**Authors:** Salima Lalani, Joseph Knudsen, James Kenney, Didier Hober, C. Michael DiPersio, Allen Gerber

**Affiliations:** ^1^ Department of Molecular & Cellular Physiology, Albany Medical College, Albany, NY, United States; ^2^ Department of Biological Sciences, University at Albany, State University of New York, Albany, NY, United States; ^3^ Laboratoire de Virologie ULR3610, Univ Lille, Centre Hospitalier Universitaire de Lille, Lille, France; ^4^ Department of Surgery, Albany Medical College, Albany, NY, United States; ^5^ Department of Neurology, Castle Point Medical Center, Wappingers Falls, NY, United States

**Keywords:** type 1 diabetes, coxsackievirus, microRNA, antiviral, pancreatic β cells, trophoblast cells

## Abstract

The epidemiological association of coxsackievirus B infection with type 1 diabetes suggests that therapeutic strategies that reduce viral load could delay or prevent disease onset. Moreover, recent studies suggest that treatment with antiviral agents against coxsackievirus B may help preserve insulin levels in type 1 diabetic patients. In the current study, we performed small RNA-sequencing to show that infection of immortalized trophoblast cells with coxsackievirus caused differential regulation of several miRNAs. One of these, hsa-miR-AMC1, was similarly upregulated in human pancreatic β cells infected with coxsackievirus B4. Moreover, treatment of β cells with non-cytotoxic concentrations of an antagomir that targets hsa-miR-AMC1 led to decreased CVB4 infection, suggesting a positive feedback loop wherein this microRNA further promotes viral infection. Interestingly, some predicted target genes of hsa-miR-AMC1 are shared with hsa-miR-184, a microRNA that is known to suppress genes that regulate insulin production in pancreatic β cells. Consistently, treatment of coxsackievirus B4-infected β cells with the hsa-miR-AMC1 antagomir was associated with a trend toward increased insulin production. Taken together, our findings implicate novel hsa-miR-AMC1 as a potential early biomarker of coxsackievirus B4-induced type 1 diabetes and suggest that inhibiting hsa-miR-AMC1 may provide therapeutic benefit to type 1 diabetes patients. Our findings also support the use of trophoblast cells as a model for identifying microRNAs that might be useful diagnostic markers or therapeutic targets for coxsackievirus B-induced type 1 diabetes.

## 1 Introduction

Type 1 diabetes (T1D) is an autoimmune disease caused by persistent immune-mediated destruction of insulin-producing pancreatic β cells (1). It is estimated that 1/300 people in the United States develop T1D by the age of 18 years (2). Currently, there is no prevention or cure for T1D, which can only be managed with life-long insulin supplements, immunotherapies, or islet cell transplants. T1D is a complex disease, wherein a combination of genetic and environmental factors interact to trigger immune-mediated destruction of pancreatic β cells. Numerous clinical observations suggest a causal link between coxsackievirus B (CVB) infection and the onset of T1D. For example, studies have shown that if a pregnant woman is infected with CVB, her child will have increased risk of developing T1D (3, 4). Moreover, case studies have shown that the onset of diabetes often occurred during an active CVB infection (5, 6). Other studies have shown that patients with newly diagnosed T1D often have higher levels of CVB antibodies (7), or that CVB infection persists in T1D patients (8–10). Individuals whose siblings have T1D are more likely to develop T1D following a CVB infection, indicating an underlying genetic component (3).

The epidemiological association of CVB with T1D suggests that treating CVB infection to reduce viral load could delay or prevent the onset of T1D. Indeed, mounting evidence from preclinical and clinical studies supports a causal role of viral infection in triggering T1D. For example, one study showed that non-obese mice were protected from coxsackievirus B4 (CVB4)-induced T1D by treatment with a monovalent vaccine against conserved regions of viral protein 1 in CVB4 (11). The Provention Bio PRV-101 multivalent vaccine study to target CVB is currently in clinical trials (NCT04690426) for prevention of T1D (12). More recently, Krogvold and coworkers showed in a randomized phase II clinical trial that insulin levels in newly diagnosed T1D patients were preserved following treatment with antiviral agents (13). These findings indicate that antiviral strategies have potential to prevent or treat T1D. It follows that microRNAs (miRNAs) that support or promote CVB infection may represent novel targets to inhibit virus-induced T1D.

miRNAs are non-coding RNAs that can regulate gene expression through inhibition of mRNA translation (14). The relationship of miRNAs to viral immunity implicates them in CVB-mediated autoimmunity associated with T1D (15). Moreover, some miRNAs are implicated in the etiology of T1D. For example, miR-146a is differentially expressed in T1D patients and contributes to diabetic complications by regulating the inflammatory response (16). Similarly, miR-184-3p is enriched in insulin-producing pancreatic β cells where it regulates several β cell functions (17). Importantly, many miRNAs are differentially regulated in the placenta during viral infection and have been implicated in providing antiviral immunity to the fetus (18). Thus, a better understanding of the early immune response may reveal important clues regarding how response to viral infection later in life (e.g., in pancreatic β cells) may lead to T1D. In particular, trophoblast cells that comprise the epithelial cell compartment of the placenta may provide a useful model for investigating how the prenatal immune system is altered during viral infection, and how certain miRNAs are impacted by and/or alter viral infection.

Given the importance of miRNAs in regulating viral infection and their associations with T1D etiology, we hypothesized that CVBs alter the miRNA signature of pancreatic β cells, which may in turn lead to alterations in the transcriptome that contribute to the autoimmune response that triggers T1D. Such a role would implicate these miRNAs as therapeutic targets for inhibiting CVB infection or its downstream effects to delay or prevent T1D. In the current study, we show that infection of trophoblast cells with CVB4 leads to the dysregulation of several miRNAs. We further show that one of these miRNAs, hsa-miR-AMC1, is up-regulated in human pancreatic β cells (a target cell of CVBs) following CVB4 infection. Moreover, treatment of β cells with an antagomir to inhibit hsa-miR-AMC1 led to decreased CVB4 infection and increased insulin production. These findings identify hsa-miR-AMC1 as a novel miRNA that is up-regulated by CVB4 infection of pancreatic β cells where it further promotes infection and suppresses insulin secretion, suggesting that this miRNA may serve as a novel diagnostic marker and/or therapeutic target for CVB4-induced T1D.

## 2 Materials and methods

### 2.1 Cell lines

The immortalized pancreatic β cell line, EndoC-βH1, was purchased from Human Cell Design (HCD) and grown in βCOAT (HCD, Cat. # BC-120) or Matrigel-fibronectin (100 µg/mL Corning, Cat. #CLS356234 and 2µg/mL, Sigma, Cat. #F1141, respectively) (19), in OPTIβ1 medium (HCD, Cat. # OB1-100). Cells were passaged every 7 days. The immortalized human trophoblast cell line hTERT (Sw.71), was purchased from Abmgood (Cat. #T0532) and grown on dishes coated with Applied Cell Extracellular Matrix (Abmgood, Cat. #G422) in PriGrow IV medium (Abmgood, Cat. #TM004) supplemented with 10% fetal bovine serum (FBS, GeminiBio, Cat. #100-106), 1% L-glutamine (Corning, Cat. #25-005-CI), 10 mM HEPES (Sigma-Aldrich, Cat. #83264), 0.1 mM MEM non-essential amino acids (Sigma-Aldrich, Cat#M7145), 1 mM sodium pyruvate (GIBCO, Cat. #11360-070) and 1% penicillin-streptomycin (PSA, GIBCO, Cat. #15140-122). LLC-MK2 derivative cells (ATCC# CCL-7.1) or Vero cells (ATCC# CCL-81) were grown in Eagle’s Modified Essential Medium (EMEM, ATCC, Cat. #30-2003) supplemented with 10% FBS (GeminiBio, Cat. #100-106) and 1% PSA (GIBCO, Cat. #15140-122). All cells were grown at 37°C, 5% CO_2_.

### 2.2 Propagation of viruses

Coxsackievirus (CVB) serotype 4 (ATCC# VR-184) was propagated in LLC-MK2 derivative cells. CVB-E2 (diabetogenic strain) was obtained from Laboratoire de Virologie ULR3610, ULille, and CHU Lille, France. Briefly, a 70% confluent monolayer of LLC-MK2 was infected for 1 hour with CVBs at a multiplicity of infection (MOI) of 0.1 or 0.01. Inoculum was then removed and cells were washed with phosphate-buffered saline (Corning, Cat. #21-040-CV) and maintained in EMEM supplemented with 2% FBS and 1% PSA. Cytopathic effects (CPE) were monitored over 1-5 days or until 90% CPE was observed. The viruses were harvested by 3-freeze-thaw cycles and contents were collected and centrifuged at 10,000xg at 4°C for 10 minutes. The supernatant was collected, aliquoted, and stored at -80°C. Viral titration was performed using plaque assays as described (20).

### 2.3 Antagomirs

A customized antagomir against hsa-miR-AMC1 (Integrated DNA Technologies) was dissolved in DNase/RNase-free water (100 µM or 500 µM) and aliquoted for storage at -80°C.

### 2.4 RNA sequencing and bioinformatics

#### 2.4.1 Small RNA sequencing (RNA-seq)

Sw.71 cells were seeded (3x10^5^ cells/well) overnight and then infected with CVB4-JVB strain for 24 hours at a multiplicity of infection (MOI) of 1. Infected cells were washed and pelleted for RNA extraction, library preparation, and small RNA-Seq (Genewiz, Azenta). Reads were aligned to miRbase (miRNA), and differential gene expression was performed using DESeq2. For novel miRNA prediction, sequences were aligned to the human genome and subjected to RNA folding and secondary structure analysis (miRDeep2, V2_0_0_7). All experiments were performed in three biological replicates (i.e., in triplicate).

#### 2.4.2 Standard RNA-seq

Sw.71 cells (3x10^5^/well) or EndoC-βH1 cells (6.7x10^5^/well) were seeded overnight and then infected with CVB4 (CVB4-JVB or E2 strain) for 24 hours at a MOI=1 (Sw.71 cells) or for 1 hour at a MOI=0.1 (EndoC-βH1 cells). Infected cells were washed and pelleted for RNA extraction (RNeasy Plus Kit, Cat. #74136), library preparation, and RNA-Seq (Genewiz, Azenta). Reads were aligned to the human reference genome, Genome Reference Consortium Human Build 38 (21), with STAR 2.7.10b (22). Differential gene expression was performed using DESeq2 1.40.2 (23). All experiments were performed in triplicate.

### 2.5 miRNA isolation and qPCR

miRNA was extracted from infected cells using a mirVana™ miRNA isolation kit (ThermoFisher Scientific, Cat. #AM1560). Reverse transcription was performed using TaqMan™ MicroRNA Reverse Transcription Kit (ThermoFisher Scientific, Cat. #4366596) and TaqMan™ MicroRNA Assay (ThermoFisher Scientific, Cat. #4427975, assay IDs 001006 and CT9HJTV) using a custom-designed looped RT primer to produce cDNA specific to small-nucleolar RNA “RNU48” (control miRNA) or hsa-miR-AMC1, according to the manufacturer’s instruction. qPCR was performed using TaqMan™ Fast Advanced Master Mix (ThermoFisher Scientific, Cat. #4444557) in a BioRad CFX96 TouchTM Real-Time PCR Detection System. Fold-change in miRNA expression was calculated using the 2-ΔΔCT method (24). All experiments were performed in triplicate.

### 2.6 Cytotoxicity analysis

To evaluate cytotoxicity of antagomirs on EndoC-βH1 cells, the CellTiter 96 Aqueous One Solution Cell Proliferation MTS assay kit (Promega, Cat. #G3582) was used according to the manufacturer’s instructions. Briefly, 2.24x10^4^ cells/well were seeded in 96-well plates pre-coated with Matrigel-fibronectin matrix, as described above. Antagomir was serially diluted (10 µM - 0.1 µM) and transfected into EndoC-βH1 cells using OptiMEM media (Gibco, Cat. #31985062) and Lipofectamine RNAiMAX Transfection Reagent (Invitrogen, Cat. #13778150) following the manufacturer’s instructions. After 3 hours transfection mix was removed and replaced with EndoC-βH1 growth medium. Cells were incubated at 37°C for 24h, then MTS solution was added to each well and incubated for an additional 2 hours for color development. Plates were read at 490 nm and cytotoxicity was plotted using GraphPad Prism (v.9.5.1). All experiments were performed in triplicate.

### 2.7 Viral inhibition analysis

To evaluate effects of miRNA inhibition, EndoC-βH1 cells grown on 96-well plates were transfected with antagomir 24 hours before infection with CVB4-JVB or CVB4-E2 strains (MOI=0.1). After incubation for 1 hour, the inoculum was removed, and cells were incubated in EndoC-βH1 cells growth medium for another 24 hours. Supernatants were collected, and plaque assays performed to determine viral infectivity. The percentage of viral inhibition was plotted using GraphPad Prism (v.9.5.1). All experiments were performed in triplicate.

### 2.8 Glucose-stimulated insulin secretion (GSIS) analysis

GSIS was performed as described by Tang et al. (25) with minor modifications. Briefly, EndoC-βH1 cells were transfected with antagomir or mock-transfected without antagomir for 24 hours, then infected with CVB4-E2 or CVB4-JVB strain for 1 hour or left uninfected as a control. The next day cells were serum-starved, first in serum-free medium for 1 hour followed by Krebs buffer solution for 1 hour (Human Cell Design, βKREBS^®^). Cells were then stimulated with 20 µM glucose for 40 minutes, and supernatant was collected and stored at -20°C prior to enzyme-linked immunosorbent assay (ELISA) to quantify insulin (Human Insulin Kit, Mercodia, Cat. #10-1113-01). ELISA was performed on duplicate samples from each of three independent experiments.

### 2.9 Statistical analysis

Data are presented as average ± S.E.M from three biological replicates (n=3), with at least two technical replicates for each experiment. Statistical significance was determined using a t-test or one-way ANOVA, as detailed in figure legends.

## 3 Results

### 3.1 Identification of differentially expressed miRNAs in CVB4-infected trophoblast cells

To identify miRNAs that might regulate T1D-associated genes following CVB infection, we infected Sw.71 cells with a prototype/reference strain of CVB4, CVB4-JVB, for 24 hours then isolated total RNA and performed miRNA sequencing. **Table 1** shows the top 5 differentially expressed miRNAs in response to CVB4-JVB infection, compared to uninfected cells. The novel miRNAs were predicted using hairpin structures of the precursor miRNAs. The miRanda (v3.3a) target scanner was used to predict target sites based on miRNA sequences and corresponding genomic cDNA sequences. As an example, predicted target genes of hsa-miR-AMC1 are listed in **Supplementary Table S1**.

**Table 1:** Top 5 differentially expressed miRNAs in CVB4-infected trophoblast (Sw.71) cells (n=3).

miRNA	log fold change	log CPM*	*p* Value	False discovery rate
hsa-miR-1304-5p	-10.25544649	2.040398839	1.28E-06	0.003426258
hsa-miR-3913-5p	9.516579482	1.324338283	1.01E-05	0.013449967
hsa-miR-AMC1	14.00786084	5.758995884	1.89E-05	0.014947595
hsa-miR-AMC2	13.7003916	5.452149213	2.24E-05	0.014947595
hsa-miR-AMC3	9.279520905	1.097630449	3.70E-05	0.019778888

*Log CPM = logarithm of counts per million reads.

### 3.2 hsa-miR-AMC1 is expressed in pancreatic β cells

**Figure 1:**
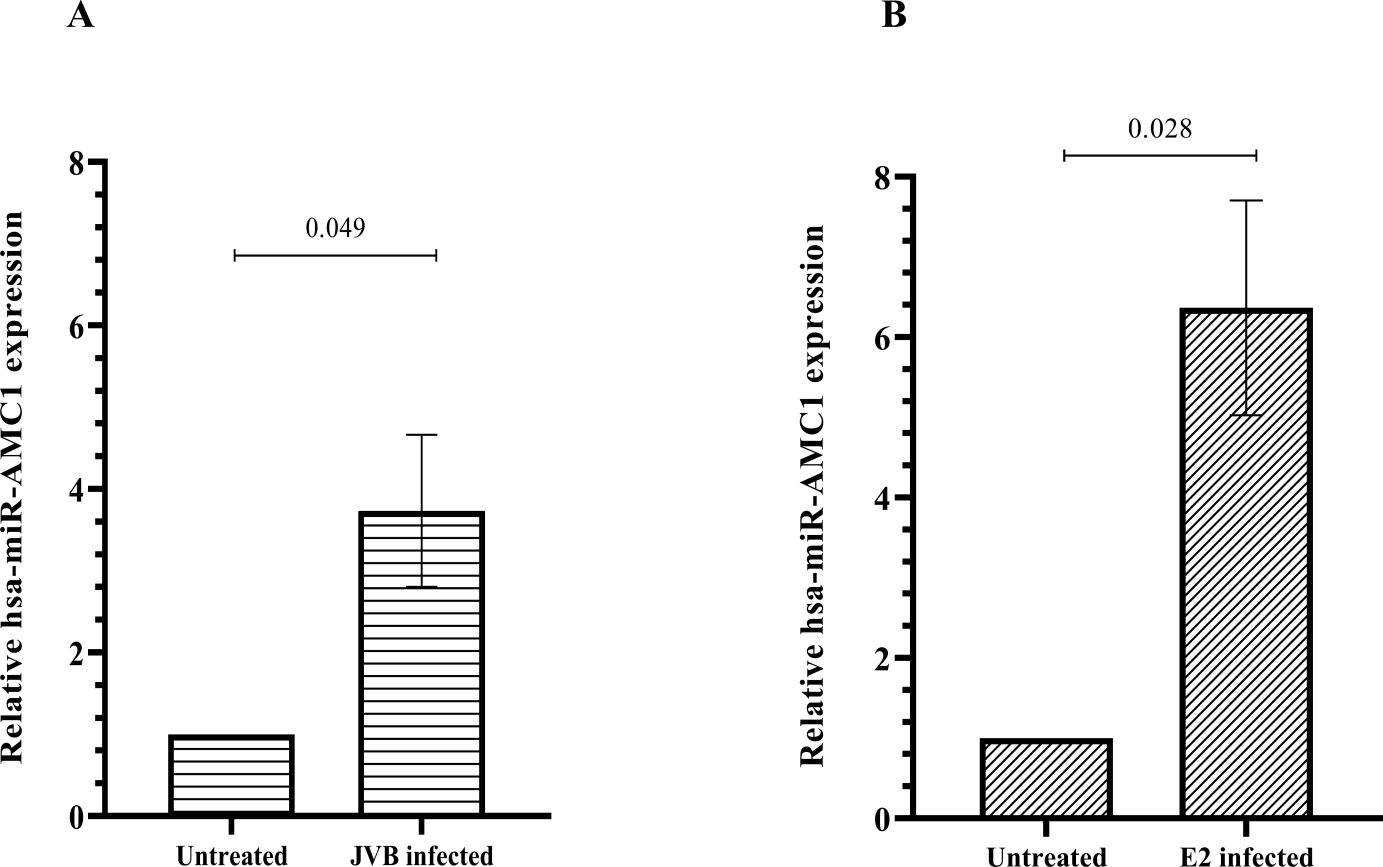
hsa-miR-AMC1 is upregulated in pancreatic β cells following CVB4 infection. Cells were left uninfected or were infected with the JVB strain **(A)** or the E2 strain **(B)** of CVB4, as indicated. qPCR was performed to determine relative hsa-miR-AMC1 expression after normalization to RNU48 expression (a small-nucleolar RNA control). For **(A, B)** hsa-miR-AMC1 expression in uninfected cells is set at a value of 1.0. Data are average ± S.E.M; t-test, *p* values are indicated, n = 3 biological replicates.

We chose to further investigate hsa-miR-AMC1 since this novel miRNA has not been explored in any disease context. Interestingly, pathway enrichment analysis of predicted hsa-miR-AMC1 target genes (**Supplementary Table S1**) revealed a 5.6-fold enrichment of predicted hsa-miR-184 target genes (false discovery rate, 4.7E-02). This finding is intriguing, as hsa-miR-184 is expressed in pancreatic β cells where it negatively regulates genes involved in insulin production, some of which are involved in T1D (26–28). To determine whether hsa-miR-AMC1 is also expressed in pancreatic β cells, and whether its expression is altered by CVB4 infection, we performed qRT-PCR of uninfected cells and cells infected with the prototype CVB4-JVB strain (**Figure 1A**) or a clinical diabetogenic variant, CVB4-E2 (**Figure 1B**). The results showed that not only is hsa-miR-AMC1 expressed in pancreatic β cells, but it is also up-regulated during infection with either CVB4 strain (**Figures 1A, B**).

### 3.3 Inhibition of hsa-miR-AMC1 reduces CVB4 infection of pancreatic β cells

**Figure 2:**
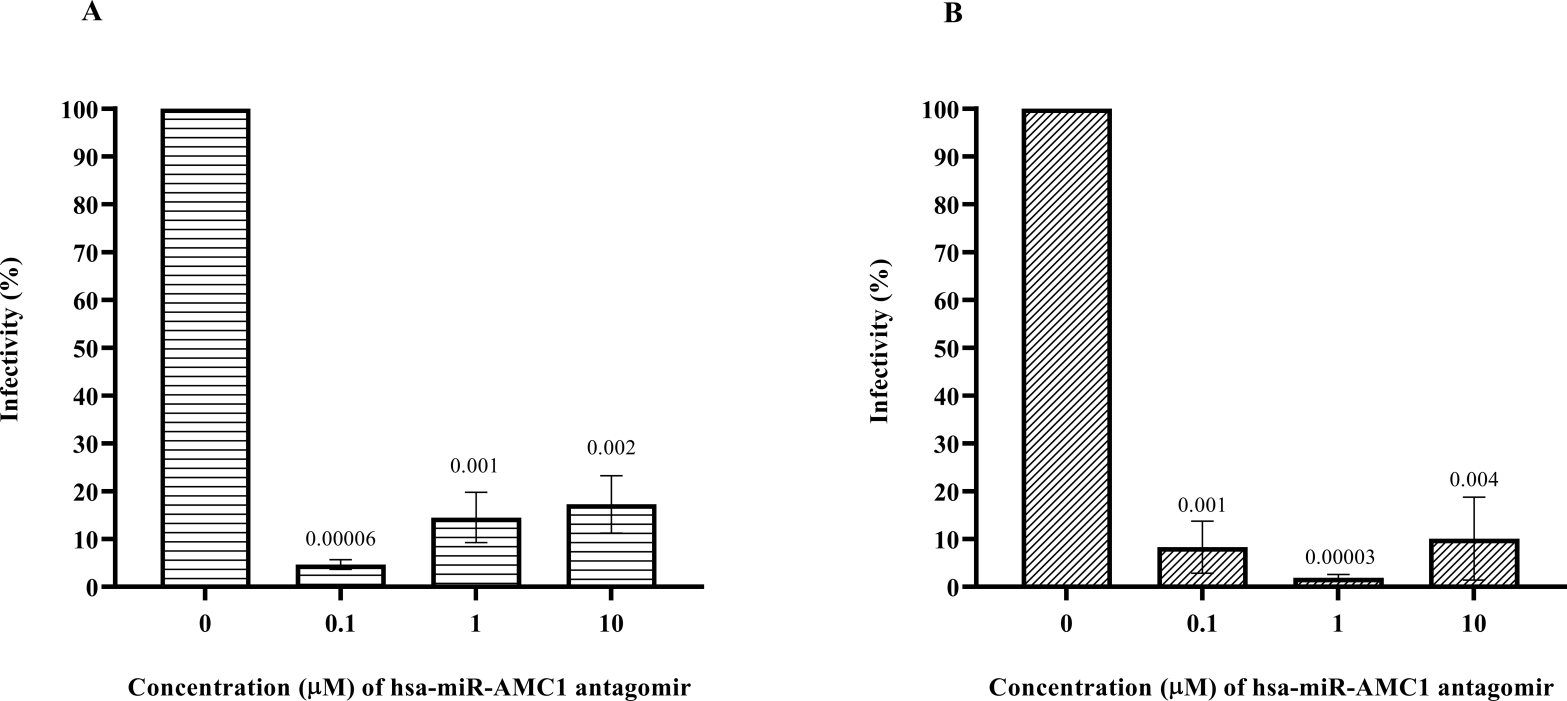
Inhibition of pancreatic β cell infection by **(A)** CVB4-JVB or **(B)** CVB4-E2 following treatment with non-cytotoxic concentrations of hsa-miR-AMC1 antagomir, determined by plaque assays using LLCMK2 cells. For **(A, B)** infectivity in the absence of antagomir is set at 100%. Data are average ± S.E.M.; n = 3 biological replicates; *p* values are indicated, One-way ANOVA followed by *post hoc* Tukey’s multiple comparisons test.

Pancreatic β cells are a known site of postnatal CVB4 infection and replication (10, 29). Since we observed that hsa-miR-AMC1 was highly up-regulated in CVB4-infected cells (**Table 1**; **Figure 1**), we next evaluated the effect of a hsa-miR-AMC1 inhibitor (antagomir) on CVB4 infectivity of β cells. A cell viability assay showed that treatment with the hsa-miR-AMC1 antagomir was well tolerated by pancreatic β cells over a range of concentrations (0.1 µM to 10 µM) (**Supplementary Figure S1**). Interestingly, treatment with the hsa-miR-AMC1 antagomir significantly inhibited infection of pancreatic β cells (p <0.0001) by either the prototype CVB4-JVB strain (**Figure 2A**) or the diabetogenic CVB4-E2 variant (**Figure 2B**). Taken together, our findings indicate that hsa-miR-AMC1 is induced by CVB4 infection in pancreatic β cells and promotes CVB4 infection. We speculate that the initial induction of hsa-miR-AMC1 upon CVB4 infection may lead to altered regulation of target genes that drive a positive feedback loop to enhance infectivity.

### 3.4 Inhibition of hsa-miR-AMC1 impacts insulin production in pancreatic β cells infected with the diabetogenic CVB4-E2 strain

**Figure 3:**
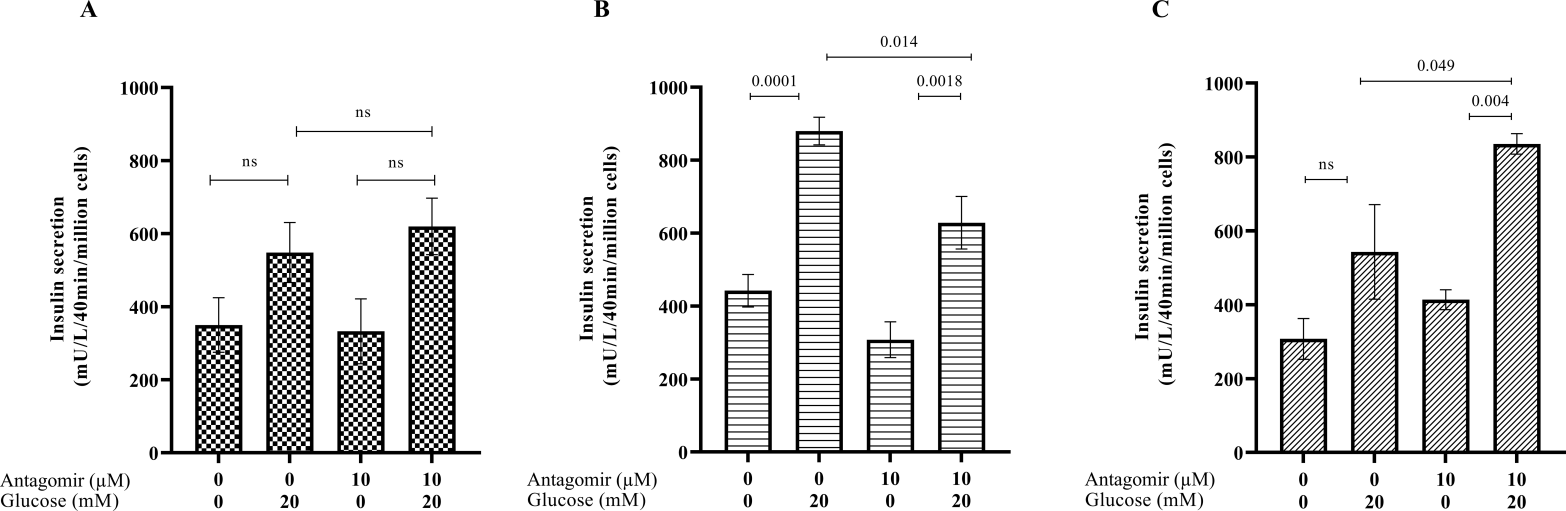
Effects of hsa-miR-AMC1 antagomir on glucose-stimulated insulin secretion (GSIS) in **(A)** non-infected, **(B)** CVB4-JVB-infected, and **(C)** CVB4-E2-infected pancreatic β cells. Data are average ± S.E.M.; n = 3 biological replicates; *p* values are indicated, ns, non-significant, One-way ANOVA followed by *post hoc* Tukey’s multiple comparisons.

We next determined if treating pancreatic β cells with the hsa-miR-AMC1 antagomir alters insulin secretion in a high-glucose environment, without and with CVB4 infection. We first determined insulin production in the absence or presence of antagomir without infection. One-way ANOVA showed that insulin production was increased when cells were challenged with 20 µM glucose, with or without antagomir (p = 0.0484, F = 3.133). Tukey’s HSD test for multiple comparisons revealed no significant difference in this induction between these two groups (p > 0.05, **Figure 3A**), suggesting that antagomir treatment had no appreciable effect. We next treated cells with antagomir followed by infection with CVB4-JVB or CVB4-E2. In CVB4-JVB infected cells without antagomir, we observed the expected trend of increased insulin upon glucose challenge (p = 0.0001). This increase remained significant in antagomir-treated cells (p = 0.0018), although it appeared dampened (**Figure 3B**). In contrast, when cells were infected with the diabetogenic CVB4-E2 variant under high glucose conditions, insulin levels were increased by antagomir treatment when compared with untreated cells (p = 0.049, **Figure 3C**). We speculate that this effect of antagomir-treatment on insulin levels could be due to either protection of pancreatic β cells from virus-induced cell death or increased ability of cells to secret insulin. In either case, these results suggest that hsa-miR-AMC1 inhibition in diabetogenic CVB4 strains (e.g., E2 strain) might lead to altered gene expression that controls glucose-stimulated insulin levels.

### 3.5 Identification of potential hsa-miR-AMC1 gene targets

We used the miRanda algorithm (v3.3a) to identify 64 genes with predicted target sites for hsa-miR-AMC1. Intriguingly, this hsa-miR-AMC1 target gene set (**Supplementary Table S2**) indicated enrichment of the “Insulin/IGF-MAPK cascade” (Enrichr, Panther 2016) (30–32), which regulates carbohydrate metabolism and insulin-like growth factor receptors. We then asked whether any of these genes are also differentially regulated in pancreatic β cells upon CVB4 infection. RNA-seq followed by assessment of differential gene expression using DESeq2 revealed 2739 or 132 differentially expressed genes (DEGs) following CVB4-E2 or CVB4-JVB infection, respectively (adjusted p-value < 0.05). We next determined whether any of these DEGs are predicted hsa-miR-AMC1 target genes. Interestingly, only one gene, leucine-rich repeat LGI family member 3 (LGI3), was common among all groups (**Supplementary Figure S2A**). Intriguingly, LGI3 is a prognostic marker of pancreatic cancer and regulates a number of relevant genes (33). For example, LGI3 regulates insulin-like growth factor binding protein 5 (IGFBP5), which is downregulated in T1D (34) and is associated with CVB infection in patient samples (35). Thus, LGI3 may be an interesting hsa-miR-AMC1 target gene for future studies. A heat map shows the expression profile of the thirteen genes that were predicted target genes of hsa-miR-AMC1 and also differentially expressed in pancreatic β cells infected with the diabetogenic CVB4-E2 strain (**Supplementary Figure S2B**). Of note, some of these genes have roles in regulating glucose metabolism and insulin regulation (36–38).

## 4 Discussion

Extensive research has revealed the multifactorial etiology of T1D. Genetic factors such as specific HLA types play a major role in a child’s susceptibility to T1D, and the risk of getting T1D increases 15-fold with a family history, particularly when the father has T1D (39). Importantly, however, ~80% of T1D patients do not have a family history of T1D, indicating that environmental factors play a major role in the disease etiology (2). Certain viruses are major suspects in triggering autoimmune conditions such as multiple sclerosis, rheumatoid arthritis, lupus erythematosus. Indeed, mounting evidence implicates enteroviruses such as CVB as likely triggers of the autoimmune response that leads to T1D (40–42).

miRNAs are endogenous small RNAs that can regulate gene expression and are often differentially expressed when exposed to a viral infection, contributing to dysregulation of immune function and prevention of an effective interferon response (43). Enteroviruses, including CVBs, can modulate expression of miRNAs, and miRNA modulation may be a vital step in successful viral infection (44–46). While it is unclear how CVB4 regulates hsa-miR-AMC1 expression, a study of CVB3-induced viral myocarditis showed that miR-222 expression was promoted by a complex that includes Dicer and the adenosine deaminases acting on RNA (ADAR) enzyme, ADAR1p150 (47). Importantly, some miRNAs can regulate expression of genes that have been implicated in the T1D autoimmune response (48, 49). In addition, viral infection can alter miRNA pathways to facilitate viral replication (50). For example, miR-146a was significantly upregulated during CVB3-infected mice and potentially modulated TLR3 and TRAF6 genes that control β cell activity in diabetes-induced inflammation (51–53). Dysregulation of miRNAs can also hamper the function of interferons, particularly in persistent infections leading to the T1D autoimmune response (29).

In the current study, we report two known and three novel miRNAs that are differentially expressed during CVB4 infection of trophoblast cells. hsa-miR-1304-5p was down-regulated during infection, while the other four miRNAs were significantly up-regulated. Interestingly, five target genes of miRNA hsa-miR-3913 (TLR8, SIRPG, TLR7, FUT2, and SMARCE1) have been identified as risk factors of T1D, as documented in Genome-wide association studies (GWAS). Moreover, all five of the latter genes were directly or indirectly associated with an immune response to viral infection (54–57). Interestingly, hsa-miR-AMC3 has a predicted target gene, CTLA4, wherein a single nucleotide polymorphism shows strong association with T1D risk due to autoimmune susceptibility (58).

From among the several miRNAs that we determined to be altered by CVB infection, we chose to further investigate hsa-miR-AMC1 because (1) this novel miRNA has not yet been explored in any disease context, and (2) our pathway enrichment analysis for predicted hsa-miR-AMC1 target genes revealed overlap with target genes of hsa-miR-184, a known negative regulator of genes that control insulin in pancreatic β cells (26). Furthermore, enriched transcription factor and protein-protein interactions analysis of hsa-miR-AMC1 identified association with nuclear factor, erythroid 2 (NFE2), a known regulator of the heme oxygenase 1 (HMOX1) gene (59) for which down-regulation is associated with delayed onset of T1D (26), as well as with nuclear respiratory factor 1 (Nrf1), for which decreased function is associated with loss of β cell activities (60).

We identified thirteen predicted target genes of hsa-miR-AMC1 that were also differentially expressed by pancreatic β cells infected with the diabetogenic CVB4-E2 strain (**Supplementary Figure S2A**-**Supplementary Table S2**). Of these, TNS3, MAFK, GALNT10, UBE3B, and C1orf21 are also predicted target genes of hsa-miR-184 (61). This subset of thirteen genes enriched the insulin/IGF-MAPK cascade in Panther pathways. Furthermore, most of these genes were down-regulated with exception of SLC7A5, MAFK, TNS3, and ZNF707 (**Supplementary Figure S2B**). Notably, an increase in transcription factor MAFK has been identified to hamper pancreatic beta cell function (38).

To understand the potential link between CVB infection and insulin regulation, we assessed overlap/interaction between pathways regulated by CVB and insulin regulatory pathways. Indeed, CVB viruses hijack several host pathways such as phosphoinositide-3-kinase–protein kinase B/Akt (PI3K/Akt) pathway, and mitogen-activated protein kinase (MAPK) to gain a replicative advantage. The MAPK pathway controls carbohydrate metabolism and cell growth via insulin receptors and insulin-like growth factor receptors, respectively. The MAPK pathway could be initiated via enteroviral modulation of the PI3K/Akt pathway, which is used by enteroviruses in the early stages of infection to promote viral replication and decrease apoptosis (62, 63). On the other hand, AMP-activated protein kinase (AMPK) is a well-identified target for diabetes and diabetes-related symptoms (64–66) and a kinase regulator of energy homeostasis (67). AMPK is necessary for activation of Akt (68) and is modulated by viruses during an infection (69). One hypothesis is that under mitochondrial stress caused by enterovirus infection there is increased viral replication and inhibition of IFN immune response via downstream AMPK targets. Considering that hsa-miR-AMC1 may regulate the insulin/IGF pathway-MAPK cascade in pancreatic β cells infected with CBV, it is interesting to speculate that its antagomir could prevent collapse of this cascade. Consistently, we observed in cells infected with the clinical diabetogenic CVB4-E2 variant that treatment with the hsa-miR-AMC1 antagomir increased glucose-dependent induction of insulin above the level of induction in untreated cells (**Figure 3**).

Although the exact mechanism through which hsa-miR-AMC1 promotes CVB infection is not yet elucidated, some viruses utilize host miRNAs to modulate viral restriction factors. One example is miR-376b-3p, which is up-regulated during porcine reproductive and respiratory syndrome virus (PRRSV) infection. Indeed, miR-376b-3p was found to target TRIM22, a virus restriction factor that interacts with the N protein of PRRSV, thereby limiting the activity of TRIM22 to exert its antiviral defense and providing a replicative advantage to the virus (70).

Finally, our use of the trophoblast cell model is an exciting development that may have important utility in the research of CVB-induced-T1D. Indeed, there has been recent interest in investigating how maternal T1D impacts placental function and fetal development (71). Our finding that the trophoblast miRNA, hsa-miR-AMC1, is also present in pancreatic β cells and was significantly upregulated during CVB4 infection leads us to propose that the trophoblast cell model may be useful for discovering novel biomarkers with postnatal relevance to T1D.

In summary, our study has identified a novel miRNA, hsa-miR-AMC1, the inhibition of which reduces CVB4 infection of pancreatic β cells and may also increase insulin production by pancreatic β cells infected with certain diabetogenic CVB4 strains. These findings are timely, as there has been increased evidence that miRNAs play a crucial role in regulating genes implicated in various autoimmune conditions. Indeed, recent literature suggests that miRNAs could be explored as potential biomarkers (72) and as targets to manage autoimmunity (73). To explore utility of hsa-miR-AMC1 as an early biomarker of CBVB-induced T1D, future studies will assess samples obtained through consortiums such as the network for pancreatic organ donors (nPOD), environmental determinants of islet autoimmunity (ENDIA), and the environmental determinants of diabetes in the young (TEDDY). Further studies are also warranted to elucidate mechanisms of hsa-miR-AMC1 action toward exploiting it as a novel therapeutic target.

## Data Availability

The data presented in the study are deposited in the Gene Expression Omnibus (GEO) repository, accession number GSE278756.
