# Supplementary material for: A novel microRNA promotes coxsackievirus B4 infection of pancreatic β cells
Source: Front Immunol. 2024 Dec 4;15:1414894. doi: 10.3389/fimmu.2024.1414894 (PMC11652211; doi:10.3389/fimmu.2024.1414894)
Supplement: Supplementary file 1 [file DataSheet1.docx]

**
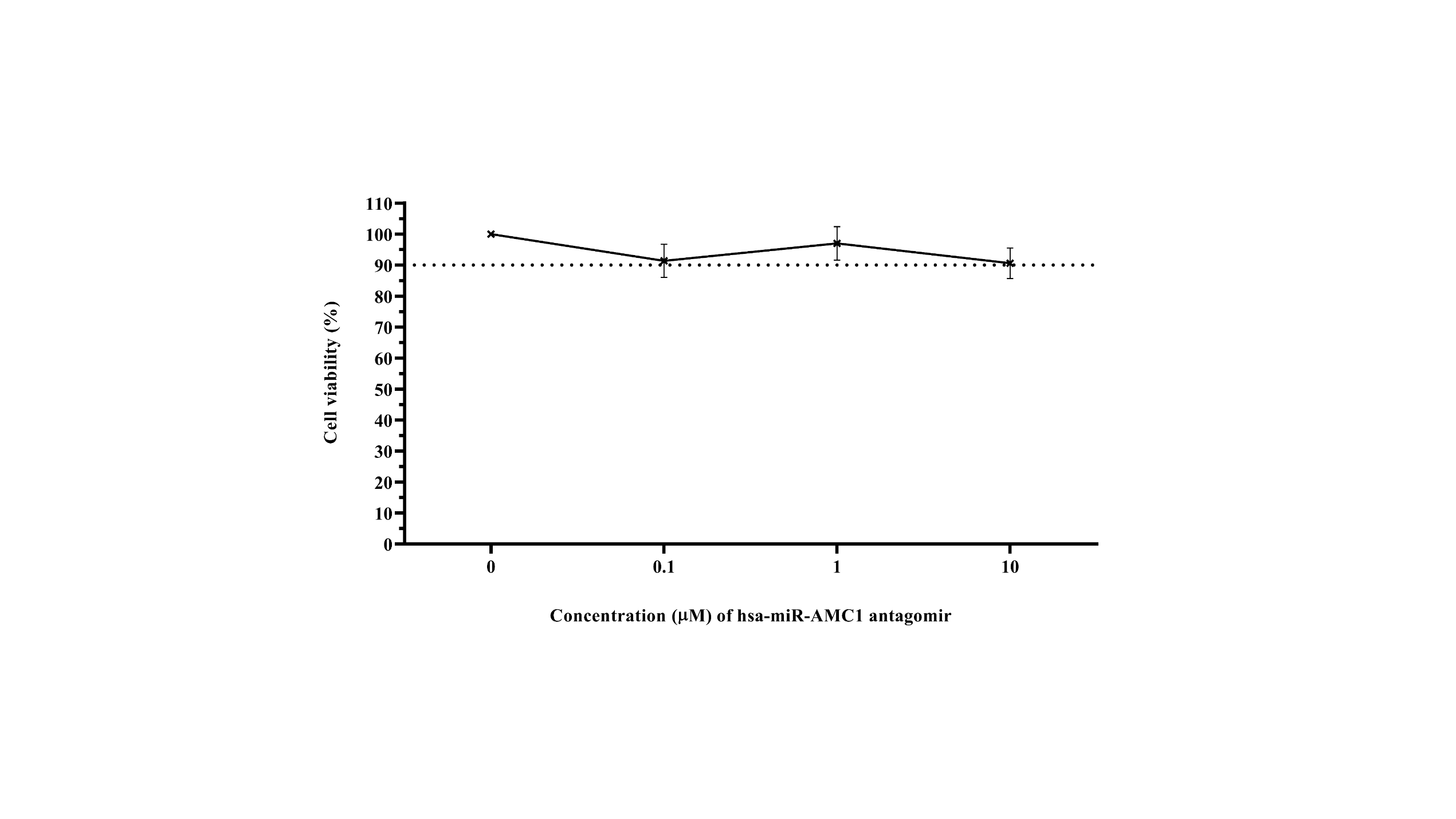
**

**Supplementary Figure 1:** Antagomir against hsa-miR-AMC1 is not cytotoxic in pancreatic β cells. Cell viability was determined using an MTS assay to measure absorbance at 490 nm with a microplate reader. The dotted line represents the 90% viability cutoff criteria. Data are presented as average viability ± S.E.M from three biological replicates.
