# Supplementary material for: A novel microRNA promotes coxsackievirus B4 infection of pancreatic β cells
Source: Front Immunol. 2024 Dec 4;15:1414894. doi: 10.3389/fimmu.2024.1414894 (PMC11652211; doi:10.3389/fimmu.2024.1414894)
Supplement: Supplementary file 2 [file DataSheet2.docx]

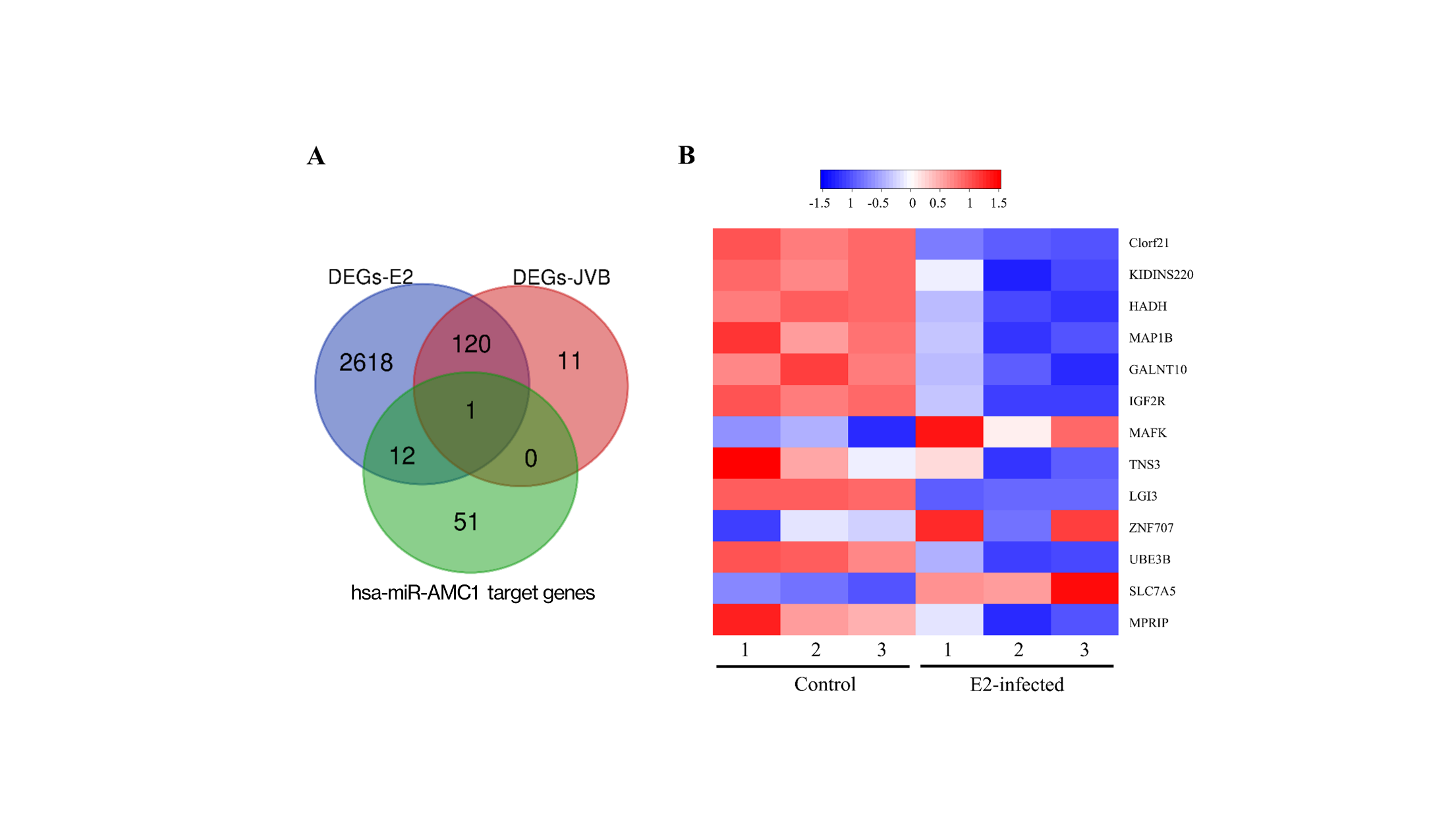


**Supplementary Figure 2**. **(A)** Venn diagram of common hsa-miR-AMC1 predicted target genes and differentially expressed genes (DEGs) in CVB4-E2 and CVB4-JVB infected pancreatic β cells and **(B)** heatmap of common hsa-miR-AMC1 predicted target genes and genes differentially expressed in CVB4-E2 infected pancreatic β cells (n=3). Blue represents downregulated genes; red represents upregulated genes.
