# Supplementary material for: A novel microRNA promotes coxsackievirus B4 infection of pancreatic β cells
Source: Front Immunol. 2024 Dec 4;15:1414894. doi: 10.3389/fimmu.2024.1414894 (PMC11652211; doi:10.3389/fimmu.2024.1414894)
Supplement: Supplementary file 3 [file Table1.docx]

**Supplementary Table 1**: Predicted hsa-miR-AMC1 target genes.

| LGI3 | ZNF117 | CROCCP3 | SMYD3 |
| --- | --- | --- | --- |
| PSMA2 | HADH | ZNF707 | CFAP410 |
| ATOX1 | PRR23B | KRT17P2 | UBE3B |
| WNT3A | CYHR1 | CLASRP | PLRG1 |
| MPRIP | ING1 | ZMIZ1 | FAM86EP |
| MFSD2B | TBC1D10B | SCAMP4 | GALNT10 |
| OR51A9P | SEC16A | IL2RB | ARMS2 |
| MAP1B | OR52H1 | KIDINS220 | FAM86DP |
| ARFGEF3 | ATCAY | RPS6P14 | OR2T5 |
| PLPP2 | COL20A1 | SHROOM2 | C1orf21 |
| HOOK2 | SLC2A4RG | MTND5P5 | OCSTAMP |
| ITPKB | TNS3 | KCNQ3 | OR2T29 |
| MAFK | PAQR5 | TNK1 | PPP1R16A |
| ACTG2 | NOC4L | IHO1 | CA5A |
| ATP13A1 | IGSF9B | SLC7A5 | IGF2R |
| SUPT20H | RAB8A | MED15P7 | MISP3 |
