# Supplementary material for: A novel microRNA promotes coxsackievirus B4 infection of pancreatic β cells
Source: Front Immunol. 2024 Dec 4;15:1414894. doi: 10.3389/fimmu.2024.1414894 (PMC11652211; doi:10.3389/fimmu.2024.1414894)
Supplement: Supplementary file 4 [file Table2.docx]

**Supplementary Table 2**: Predicted hsa-miR-AMC1 target genes that are differentially expressed in CVB4-E2 infected pancreatic β cells versus uninfected cells.

| Symbol | log2FoldChange | p-adj (False Discovery Rate) |
| --- | --- | --- |
| SLC7A5 | 0.908602 | 5.48E-13 |
| HADH | -0.42553 | 3.21E-10 |
| LGI3 | -1.50117 | 1.71E-09 |
| IGF2R | -0.34264 | 6.22E-07 |
| UBE3B | -0.50107 | 1.95E-06 |
| MAP1B | -0.30186 | 1.43E-05 |
| MAFK | 1.064973 | 1.45E-05 |
| KIDINS220 | -0.25999 | 0.001585 |
| GALNT10 | -0.42262 | 0.002087 |
| TNS3 | 0.333692 | 0.002901 |
| MPRIP | -0.27203 | 0.014473 |
| ZNF707 | 0.55343 | 0.022207 |
| C1orf21 | -0.33416 | 0.023079 |
